# Comparison of MetaMap and cTAKES for entity extraction in clinical notes

**DOI:** 10.1186/s12911-018-0654-2

**Published:** 2018-09-14

**Authors:** Ruth Reátegui, Sylvie Ratté

**Affiliations:** 1 École de technologie supérieure, Montreal, Canada; 2 Universidad Técnica Particular de Loja, Loja, Ecuador

**Keywords:** cTAKES, MetaMap, UMLS, Clinical documents

## Abstract

**Background:**

Clinical notes such as discharge summaries have a semi- or unstructured format. These documents contain information about diseases, treatments, drugs, etc. Extracting meaningful information from them becomes challenging due to their narrative format. In this context, we aimed to compare the automatic extraction capacity of medical entities using two tools: MetaMap and cTAKES.

**Methods:**

We worked with i2b2 (Informatics for Integrating Biology to the Bedside) Obesity Challenge data. Two experiments were constructed. In the first one, only one UMLS concept related with the diseases annotated was extracted. In the second, some UMLS concepts were aggregated.

**Results:**

Results were evaluated with manually annotated medical entities. With the aggregation process the result shows a better improvement. MetaMap had an average of 0.88 in recall, 0.89 in precision, and 0.88 in F-score. With cTAKES, the average of recall, precision and F-score were 0.91, 0.89, and 0.89, respectively.

**Conclusions:**

The aggregation of concepts (with similar and different semantic types) was shown to be a good strategy for improving the extraction of medical entities, and automatic aggregation could be considered in future works.

## Background

Electronic Health Records (EHR) or Electronic Medical Records (EMR) save patients’ information in a format that is either structured (e.g., diagnosis codes, laboratory results, medication) or unstructured (e.g., clinical notes). Clinical notes, such as discharge summaries, radiology notes, and progress notes, have an unstructured format with a narrative style. These documents provide a more complete portrait of the patient’s health [1–3], as well as additional valuable information (e.g., diagnosis, symptoms, medical history, social history, medication, lab tests, treatments, etc.). Unfortunately, unstructured formats complicate information extraction. First, they contain many abbreviations, acronyms, and specialized terms [4]. Secondly, a variety of natural languages are used, depending on the particular health professional or institution [5], and may not correspond to a general domain. Furthermore, manual annotations and analysis present in clinical notes can transform extraction into a time-consuming, labor-intensive, and error-prone endeavor [6].

Nowadays, various tools exist for extracting information from clinical texts created in an unstructured format. Two such tools, which are widely used and known in the biomedical field, are MetaMap and cTAKES [7, 8]. MetaMap was developed by the National Library of Medicine (NLM) to map biomedical text to concepts in the Unified Medical Language System (UMLS) [9, 10]. The tool uses a hybrid approach combining a natural language processing (NLP), knowledge-intensive approach and computational linguistic techniques [10]. The Clinical Text Analysis and Knowledge Extraction System (cTAKES) combines rule-based and machine learning techniques to extract information from a clinical text [6]. cTAKES executes some components in sequence to process the clinical text. Both MetaMap and cTAKES use the Unified Medical Language System (UMLS) to extract and standardize medical concepts.

The extraction of medical entities (e.g., diseases, treatments, drugs, etc.) is important for patients and medical research [4, 7, 11]. Moreover, these medical entities form the basis for other tasks such as disease correlation [1], disease classification [12, 13], disease diagnosis [5, 14], phenotype identification [2, 3] etc.

Given the significance of medical entity extraction, this paper aims to compare this extraction carried out using two different tools (MetaMap and cTAKES). For this project, we worked with the i2b2 (Informatics for Integrating Biology to the Bedside) Obesity Challenge data. The automated extraction was evaluated against the experts’ manual annotations of 14 obesity comorbidities (simultaneous presence of two chronic diseases or conditions in a patient) from discharge summaries.

## Methods

### Dataset

The i2b2 2008 Obesity dataset consists of 1237 discharge summaries of overweight and diabetic patients [15]. The documents contain two different expert annotations: textual and intuitive. In this work, we use textual annotations where experts classified 15 obesity comorbidities based on the explicit information in discharge summaries. The diseases had four classifications:

- Present: The patient has/had the disease.
- Absent: The patient does not/did not have the disease.
- Questionable: The patient may have the disease.
- Unmentioned: Absence of information of the disease in the discharge summary.

The first column of Table 1 shows the 14 comorbidities used. Hypertriglyceridemia was excluded due to a lack of sufficient samples. Out of 1237 summaries, we selected the 412 summaries which had obesity as a comorbidity.

**Table 1:** List of entities or concept

Entities annotated by experts	Entities in the first experiment	Entities or groups in the second experiment
Name of disease	Preferred name, CUI, Semantic Type	Preferred name, CUI, Semantic Type
Hypertension	Hypertensive disease, C0020538, dsyn	Hypertensive disease, C0020538, dsyn
Diabetes	Diabetes mellitus, C0011849, dsyn	Diabetes mellitus, C0011849, dsyn Diabetes mellitus, insulin-dependent, C0011854, dsyn Diabetes mellitus, non-insulin-dependent, C0011860, dsyn
Atherosclerotic Cardiovascular Disease (CAD)	Coronary artery disease, C1956346, dsyn	Coronary artery disease, C1956346, dsyn Coronary arteriosclerosis, C0010054, dsyn
Congestive Heart Failure (CHF)	Congestive heart failure, C0018802, dsyn	Congestive heart failure, C0018802, dsyn
Hypercholesterolemia	Hypercholesterolemia, C0020443, dsyn	Hypercholesterolemia, C0020443, dsyn Hyperlipidemia, C0020473, dsyn
Obstructive Sleep Apnea (OSA)	Sleep apnea obstructive, C0520679, dsyn	Sleep apnea obstructive, C0520679, dsyn
Osteoarthritis (OA)	Degenerative polyarthritis, C0029408, dsyn	Degenerative polyarthritis, C0029408, dsyn
Depression	Mental depression, C0011570, mobd	Mental depression, C0011570, mobd Depressive disorder, C0011581, mobd
Asthma	Asthma, C0004096, dsyn	Asthma, C0004096, dsyn
Gastroesophageal Reflux Disease (GERD)	Gastroesophageal reflux disease, C0017168, dsyn	Gastroesophageal reflux disease, C0017168, dsyn
Gallstones/Cholecystectomy	Cholecystectomy procedure, C0008320, topp	Cholecystectomy procedure, C0008320, topp Cholecystolithiasis, C0947622, dsyn Cholecystitis, C0008325, dsyn Cholelithiasis, C0008350, dsyn
Gout	Gout, C0018099, dsyn	Gout, C0018099, dsyn
Peripheral Vascular Disease (PVD)	Peripheral vascular diseases, C0085096, dsyn	Peripheral vascular diseases, C0085096, dsyn
Venous Insufficiency	Venous insufficiency, C0042485, dsyn	Venous insufficiency, C0042485, dsyn Postthrombotic syndrome, C0277919, patf

The second experiment grouped together some entities related to the disease annotated by the experts

*CUI* Concept Unique Identifier, *dsyn* Disease or Syndrome, *mobd* Mental or Behavioral Dysfunction, *topp* Therapeutic or Preventive Procedure, *patf* Pathologic Function

### Unified medical language system

The National Library of Medicine Unified Medical Language System (UMLS) provides terminology, coding standards, and resources for biomedical and electronic health systems. UMLS has three Knowledge Sources: the Metathesaurus, the Semantic Network and the SPECIALIST lexicon.

The Metathesaurus is organized by concepts or meanings. A concept has a unique and permanent identifier (CUI) and a preferred name. The concept is a meaning, and a meaning can have different names from different vocabularies or thesauruses. [16]. The Semantic Network provides (1) a categorization (semantic type) of all concepts represented in the UMLS Metathesaurus; and (2) a set of relationships (semantic relations) between these concepts [16]. The Semantic Network contains 133 semantic types and 54 relationships.

UMLS is based on some electronic thesauruses, classifications, code sets, and lists of controlled terms like SNOMED CT and RxNorm [16]. The Systematized Nomenclature of Medicine - Clinical Terms (SNOMED CT) is a multilingual health terminology used for the electronic exchange of clinical health information [17]. In the U.S., SNOMED CT is the national standard for electronic exchange of clinical health information [17]. On the other hand, RxNorm standardizes clinical drug names and links the names to other vocabularies used in pharmacy management and drug interaction software [18].

In this work, the medical entities extracted will be the concepts represented by the CUIs. We worked with SNOMED CT and RxNorm as vocabularies and with four semantic types (henceforth ST):

- Disease or Syndrome
- Mental or Behavioral Dysfunction
- Pathologic Function
- Therapeutic or Preventive Procedure

### Automatic extraction

We used separately MetaMap (version 2015) and cTAKES (version apache-ctakes-3.2) to extract the CUIs related with the 14 obesity comorbidities above mentioned. With each tool, two different experiments were carried out in order to extract the entities automatically.

In the first experiment, we identified one CUI code related to each comorbidity or disease. The extracted CUI and the preferred name of the concepts are shown in Table 1, column 2. In this experiment, diabetes, atherosclerotic cardiovascular disease (CAD), hypercholesterolemia, osteoarthritis, depression, venous insufficiency, and cholecystectomy have low values in the evaluation (see Table 2). Therefore, to improve the results for these diseases, a second experiment was performed.

**Table 2:** Summary of first experiment

Diseases	Number of patients			Evaluation					
	Annotations	MetaMap	cTAKES	MetaMap			cTAKES		
				Recall	Precision	F-score	Recall	Precision	F-score
Hypertension	325	336	340	0.99	0.96	0.98	0.99	0.95	0.97
Diabetes^a^	259	186	235	**0.65**	0.91	0.76	0.83	0.91	0.87
Atherosclerotic Cardiovascular Disease (CAD)^a^	181	95	199	**0.45**	0.86	0.59	0.92	0.84	0.88
Congestive Heart Failure (CHF)	172	175	183	0.89	0.87	0.88	0.92	0.86	0.89
Hypercholesterolemia^a^	172	108	92	**0.59**	0.94	0.73	**0.51**	0.95	0.66
Obstructive Sleep Apnea (OSA)	127	105	102	0.78	0.94	0.85	0.76	0.94	0.84
Osteoarthritis (OA)^a^	87	76	61	0.76	0.87	0.81	**0.67**	0.95	0.78
Depression^a^	83	105	116	0.89	**0.70**	0.79	0.99	**0.71**	0.82
Asthma	81	83	92	0.93	0.90	0.91	1.00	0.88	0.94
Gastroesophageal Reflux Disease (GERD)	76	83	85	0.97	0.89	0.93	0.99	0.88	0.93
Gallstones/Cholecystectomy^a^	74	54	58	**0.73**	1.00	0.84	0.78	1.00	0.88
Gout	56	58	58	0.98	0.95	0.96	0.98	0.95	0.96
Peripheral Vascular Disease (PVD)	37	37	32	0.97	0.97	0.97	0.84	0.97	0.90
Venous Insufficiency^a^	21	6	6	**0.29**	1.00	0.44	**0.29**	1.00	0.44
AVERAGE				0.78	0.91	0.82	0.82	0.91	0.84

The lowest values for recall and precision are in bold

^a^Disease with low evaluation

In the second experiment, we worked with two types of aggregations described below. Aggregation has been wide applied in the genetic field. For example, a pathway level is used instead of individual genes to obtain a compact representation or to improve tasks like classification or clustering [19].

**Fig. 1:**
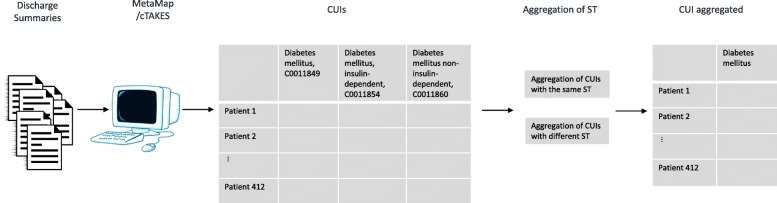
Process for the second experiment. Discharge summaries were analyzed with MetaMap or cTAKES to extract CUIs. Then some CUIs were aggregated to obtain the 14 comorbidities related with obesity

**Fig. 2:**
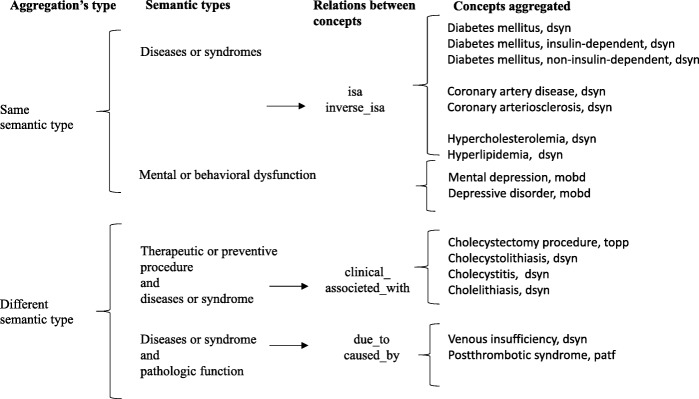
Aggregation process

1. Aggregation of CUIs with the same ST. The aggregation of CUIs belonging to the ST “Diseases or Syndromes” allowed us to cover diabetes, coronary artery disease and hypercholesterolemia, while the aggregation of CUIs belonging to the ST “Mental or Behavioral Dysfunction” allowed us to cover mental depression.
2. Aggregation of CUIs with different ST. First, we aggregated CUIs belonging to the ST “Diseases or Syndrome” with CUIs belonging to the ST “Pathologic Function”; this grouping allowed us to recover enough information to better identify venous insufficiency. Second, we aggregated CUIs belonging to the ST “Therapeutic or Preventive Procedure” with CUIs belonging to the ST “Diseases or Syndrome”; this second grouping allowed us to recover the information needed to identify cholecystectomy. Details of the CUIs grouped together are shown in Table 1, column 3. Figure 1 shows the process for the second experiment and Fig. 2 shows the aggregation process.

### Evaluation metrics

We considered the experts’ annotations as a gold standard in evaluating the automatic extraction. Only the “Present” annotation was taken into account in identifying whether the patient has or had the diseases.

We used the recall (or sensitivity), precision and F-score to evaluate the results:

1 $$ Recall=\frac{TP}{TP+ FN} $$

2 $$ Precision=\frac{TP}{TP+ FP} $$

3 $$ F\  score=2\ \frac{Precision. Recall}{\left( Precision+ Recall\right)} $$

where TP is the number of true positives of the CUIs mentioned, FN is the number of false negatives of the CUIs mentioned, and FP is the number of false positives of the CUIs mentioned.

## Results

In the first experiment (see Table 2), the averages for the recall, precision and F-score with MetaMap were 0.78, 0.91, and 0.82, respectively. With cTAKES, the averages for the same measures were 0.82, 0.91, and 0.84, respectively. MetaMap showed a low recall value for diabetes (0.65), CAD (0.45), hypercholesterolemia (0.59), and venous insufficiency (0.29). Cholecystectomy presents a satisfactory recall value (0.73) although much lower than the overall results. Also, cTAKES had low recall values for hypercholesterolemia (0.51), osteoarthritis (0.67), and venous insufficiency (0.29).

In the second experiment (see Table 3), we achieved better results. MetaMap had an average of 0.88 in recall, 0.89 in precision, and 0.88 in F-score. With cTAKES, the averages for recall, precision and F-score were 0.91, 0.89, and 0.89, respectively. That means that aggregation improves the results. For example, in the first experiment, diabetes had a recall value of 0.65 (MetaMap) and 0.83 (cTAKES), but in the second experiment, these values increased to 0.89 (MetaMap) and 0.92 (cTAKES). The same can be said about hypercholesterolemia. In the first experiment, this disease had a recall value of 0.59 (MetaMap) and 0.51 (cTAKES), but in the second experiment, these values improved to 0.88 and 0.81.

**Table 3:** Summary of second experiment

Diseases	Number of patients			Evaluation					
	Annotations	MetaMap	cTAKES	MetaMap			cTAKES		
				Recall	Precision	F-score	Recall	Precision	F-score
Hypertension	325	336	340	0.99	0.96	0.98	0.99	0.95	0.97
Diabetes^a^	259	254	266	**0.89**	**0.91**	**0.90**	**0.92**	0.89	**0.91**
Atherosclerotic Cardiovascular Disease (CAD)^a^	181	130	205	**0.60**	0.83	**0.69**	0.92	0.81	0.87
Congestive Heart Failure (CHF)	172	175	183	0.89	0.87	0.88	0.92	0.86	0.89
Hypercholesterolemia^a^	172	159	146	**0.88**	**0.96**	**0.92**	**0.81**	**0.96**	**0.88**
Obstructive Sleep Apnea (OSA)	127	105	102	0.78	0.94	0.85	0.76	0.94	0.84
Osteoarthritis (OA)	87	76	61	0.76	0.87	0.81	0.67	0.95	0.78
Depression^a^	83	109	116	**0.93**	**0.706**	**0.802**	0.99	0.71	0.82
Asthma	81	83	92	0.93	0.90	0.91	1.00	0.88	0.94
Gastroesophageal Reflux Disease (GERD)	76	83	85	0.97	0.89	0.93	0.99	0.88	0.93
Gallstones/Cholecystectomy^a^	74	65	68	**0.865**	0.99	**0.92**	**0.89**	0.97	**0.93**
Gout	56	58	58	0.98	0.95	0.96	0.98	0.95	0.96
Peripheral Vascular Disease (PVD)	37	37	32	0.97	0.97	0.97	0.84	0.97	0.90
Venous Insufficiency^a^	21	27	30	**0.905**	0.704	**0.792**	**1**	0.7	**0.824**
AVERAGE				0.88	0.89	0.88	0.91	0.89	0.89

The values improved are in bold

^a^Diseases formed by two or more UMLS concepts

CAD is a special case which illustrates the difference between both tools. For a sentence like (1) below, cTAKES recognized, among many other clues, the abbreviation “CAD”, but MetaMap did not. Consequently, the number of patients with this disease was lower in MetaMap; however, this notwithstanding, the recall increased from 0.45 to 0.6, which is a direct consequence of the aggregation of ST.

1. “Conditions, Infections, Complications, Affecting Treatment/Stay HTN, CAD, High cholesterol, OSA, OA, Depression, and Anxiety”
2. “ST depression in the inferior leads and V5-V6”
3. “was found to be in atrial flutter with a 2:1 block and 2-3 mm lateral ST depressions in V4-V6”

Depression is another interesting case. In the first experiment, it was the disease with the lowest precision in both tools, 0.70 in MetaMap, and 0.71 in cTAKES. Sentences (2) and (3) above illustrate the problem. For both sentences, MetaMap and cTAKES consider that the word “depression” refers to the disease, which is clearly not the case. In both sentences, “depression” refers to a part that is lower than the surrounding area, not to the disease. This problem increased the number of false positives. Consequently, the aggregation of ST, used in the second experiment, did not significantly increase precision. However, the aggregation of ST allowed MetaMap to increase the recall from 0.89 to 0.93.

In the first experiment, we considered the cholecystectomy procedure, but in order to know other ways to identify the presence of gallstones, we added information referring to diseases and syndromes such as cholecystolithiasis, cholecystitis, and cholelithiasis. Therefore, the second experiment increased the recall from 0.73 to 0.87 (for MetaMap), and from 0.78 to 0.87 (for cTAKES).

Venous insufficiency increased its recall from 0.29 to 0.9 (for MetaMap), and from 0.29 to 1 (for cTAKES). To improve the venous insufficiency result, we added the postthrombotic syndrome which corresponded to the ST pathologic function.

Osteoarthritis or degenerative polyarthritis presented a low recall with cTAKES, bringing us to review the automatic extraction of the disease. In many cases, health professionals use the abbreviation OA for this disease, an abbreviation which is not recognized by cTAKES; consequently, the number of patients with this disease was low as compared to MetaMap. In some cases, MetaMap mapped this disease to a precise CUI such as C0409959 (Degenerative joint disease of knee), but in other cases, when the experts classified the disease as “OA”, MetaMap and cTAKES generalized it using the general concept “arthritis”. Since osteoarthritis is a specific type of arthritis, we decided not to proceed, in that specific case, with the aggregation of all CUIs under “arthritis”.

## Discussion

Considering the results shown in Table 2 (first experiment), it is not surprising that previous authors chose to combine both tools to secure better results [20]. In this work, we avoid that combination because we intended to compare the results of each tools. The results in Table 3 (second experiment) show that at least two types of relationships have to be taken into account to obtain, with both tools, better results.

1. Aggregation of CUIs with the same ST (e.g., “Disease or Syndrome” and “Mental or Behavioral Dysfunction”): This form of aggregation takes into account the “isa/inverse_isa” relations between concepts in the Metathesaurus. This relation, allowed us to group under “diabetes mellitus”, both “insulin-dependent-diabetes” and “non-insulin-dependent-diabetes”. Similarly, “coronary arteriosclerosis” was grouped with “coronary artery disease”, “hyperlipidemia” with “hypercholesterolemia”, and “depressive disorder” with “mental depression”.
2. Aggregation of CUIs with different ST: An example here is using the Metathesaurus relation “due_to/caused_by” to combine venous insufficiency disease with the postthrombotic syndrome pathologic function. Also, we noted that for many forms of gallstones, the clinical notes mentioned the cholecystectomy procedure instead of the specific disease (e.g., cholecystolithiasis). Using the relation “clinically_associated_with”, we were able to connect the cholecystectomy procedure with the cholelithiasis disease, and then with the cholecystolithiasis and cholecystitis diseases, among others.

Tables 2 and 3 show the results of the first and second experiments. Overall, the aggregations carried out in the second experiment increased the F-score by 7.3% for MetaMap, and by 6% for cTAKES. The recall values increased by 12.8% for MetaMap and by 11% for cTAKES, while the precision values decreased slightly in both tools, − 2.2% for both MetaMap and cTAKES.

As we mentioned above, clinical notes contain many abbreviations, acronyms, and specialized terms that renders difficult the extraction of patient information. Abbreviations such as CHF and PVD were identified by both tools, but CAD and OA were not. It means that the results are sensitive to abbreviations used in the clinical notes. To resolve this problem, MetaMap allows users to define a list of abbreviations and acronyms. On the other hand, cTAKES does not have such a list [21]. In this work, we did not use any list of abbreviations with the aim to keep the same configuration for both tools, but the use of this option could help MetaMap improve its results.

In the annotations made by the experts, they used general names or maybe a preferred name to denote a comorbidity. For that reason, in the second experiment, we had to look for some UMLS concept to identify one annotated comorbidity (e.g. we matched 3 UMLS diabetes mellitus concepts). In other cases, we worked with different semantic types such as pathological function and therapeutic or preventive procedures to referred to a comorbidity mentioned by the experts (e.g. venous insufficiency and gallstones).

### Future works

In future works, we will consider the automatic aggregation of concepts or CUIs using the relations between the concepts described in the Metathesaurus and the semantic relation present in the Semantic Network.

Also, while clinical notes hold information on many medical entities, some of them are in negative contexts (e.g., “The patient does not have diabetes”). In this work, we did not use algorithms like NegEx [22] that permit a recognition of entities in negative contexts. Moreover, for the extraction of medical entities, all sections were considered, including the parts such as family history, which can describe diseases that the patient does not have. Therefore, these characteristics can be taken into account to decrease the rate of false positives and improve precision.

## Conclusion

In this paper, we compared the automatic extraction of 14 obesity comorbidities using MetaMap and cTAKES. Automatic extraction was compared to manual annotation by experts. The result of the experiments we conducted proved that cTAKES slightly outperforms MetaMap, but this situation could change considering other configuration options that each tool has such as the abbreviations list in the MetaMap tool. Moreover, we worked with two types of aggregations: aggregation of CUIs with the same semantic type and aggregation of CUIs with different semantic types. These groups improve the results. Hence, the use of cTAKES or even MetaMap, using the proposed aggregations, can represent a good strategy to replace the manual extraction of medical entities.

Finally, it should be noted that both tools are constantly improving the quality of their results. However, we believe that the combination of both, along with the aggregations, might even permit to cover complementary cases where both tools give different results.

## Abbreviations

CAD: Atherosclerotic cardiovascular disease
CHF: Congestive heart failure
CUI: Concept unique identifier
EHR: Electronic Health Record
GERD: Gastroesophageal reflux disease
OA: Osteoarthritis
OSA: Obstructive sleep apnea
PVD: Peripheral vascular disease
UMLS: Unified Medical Language System
